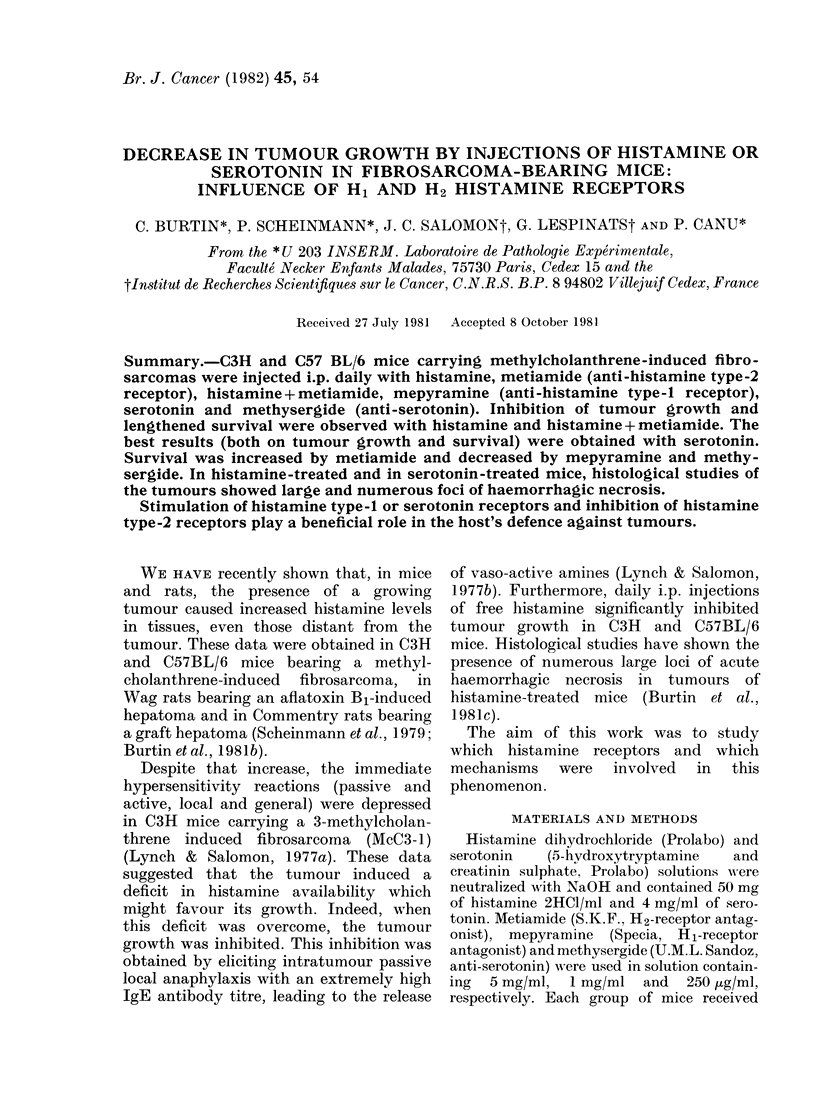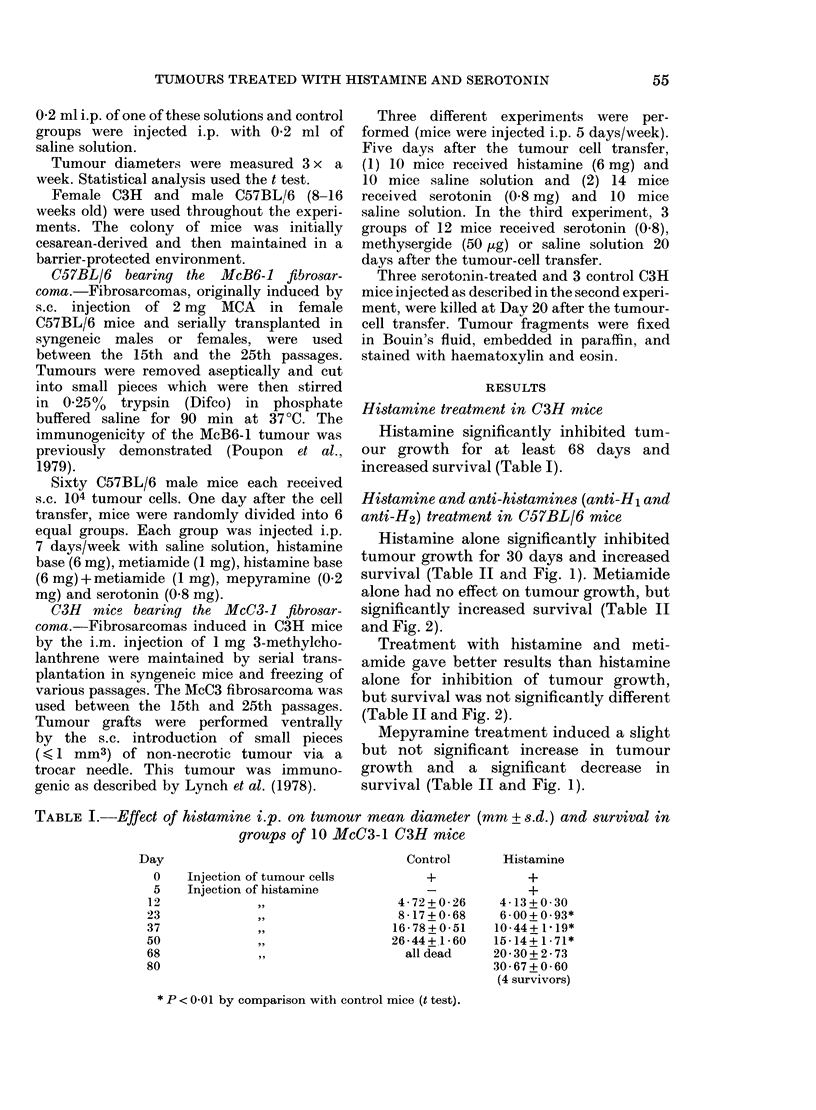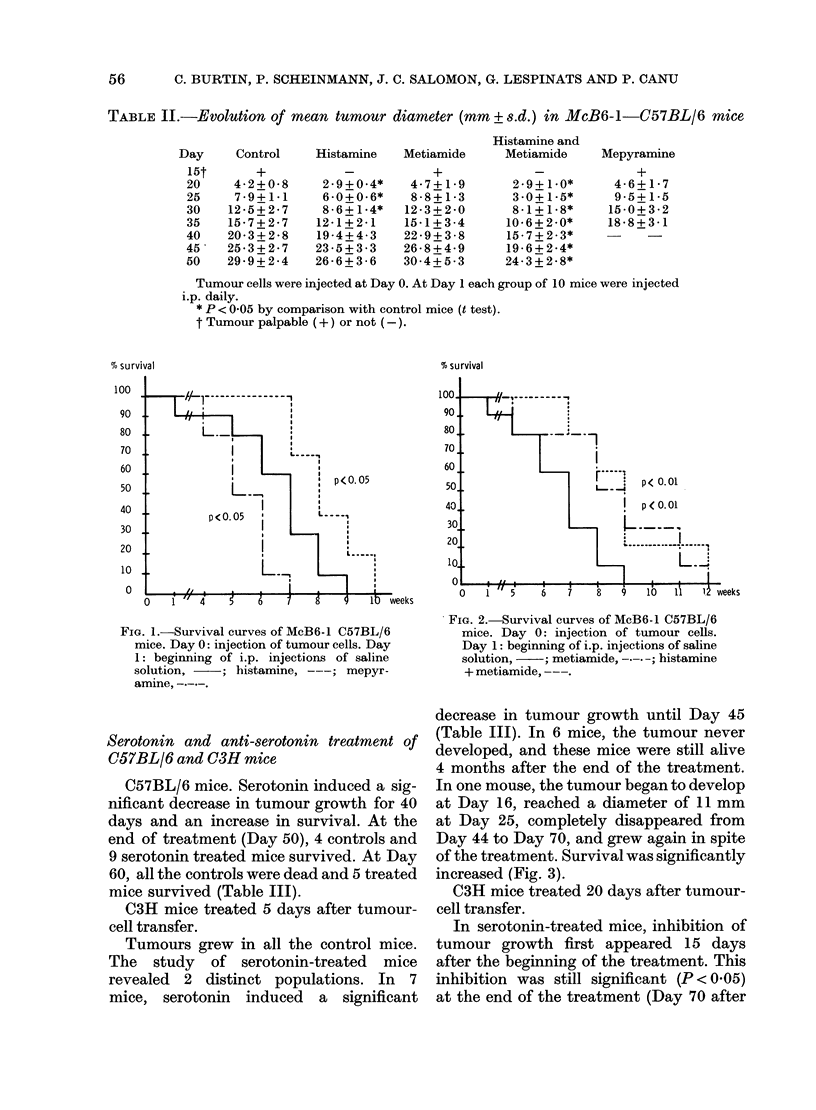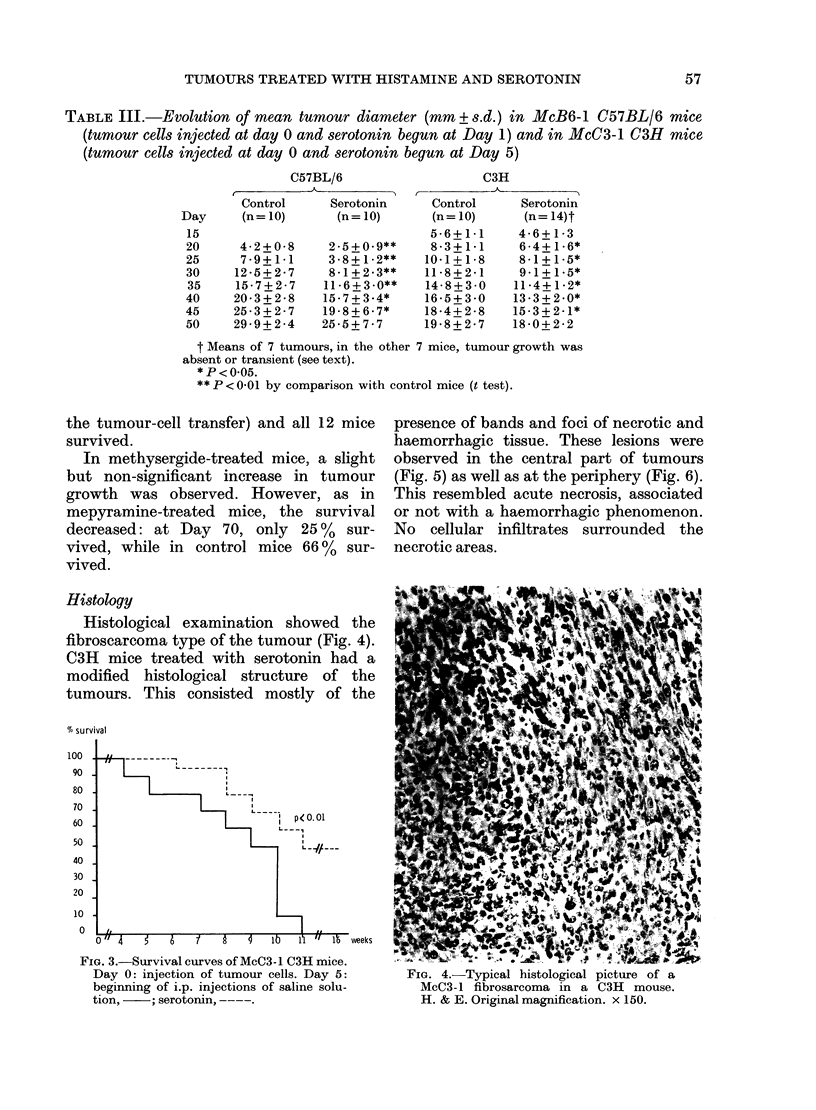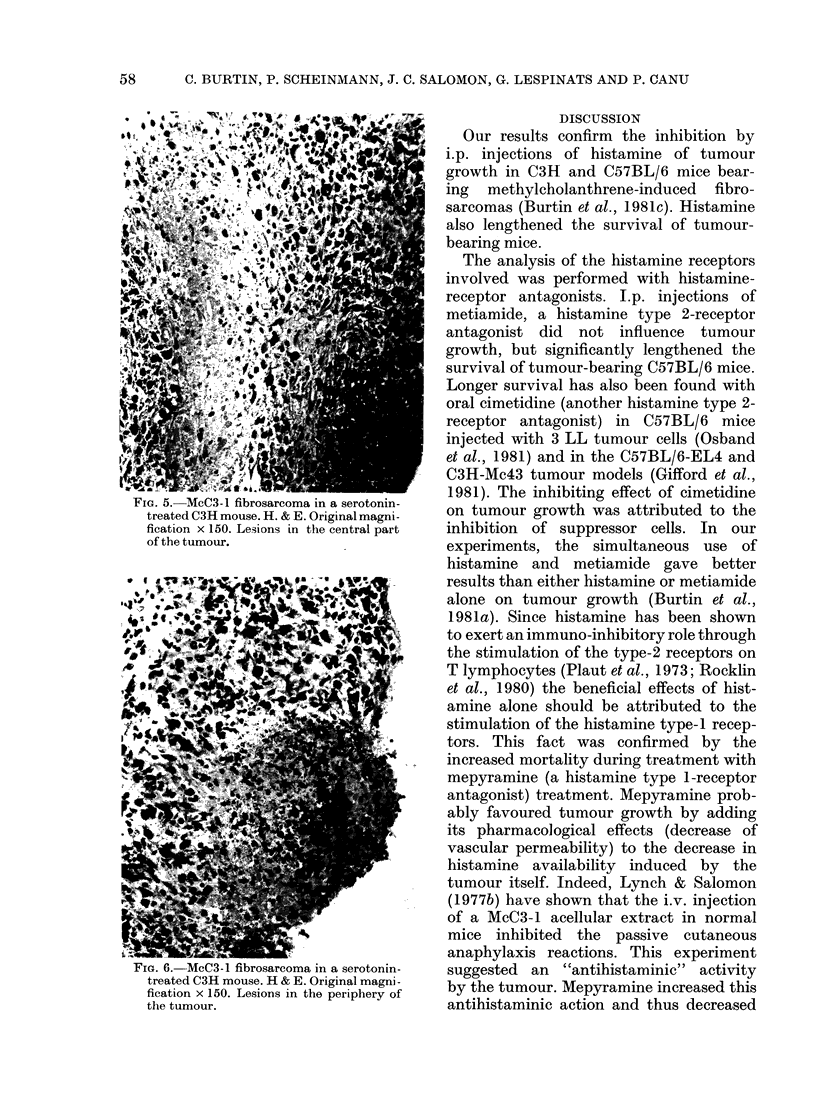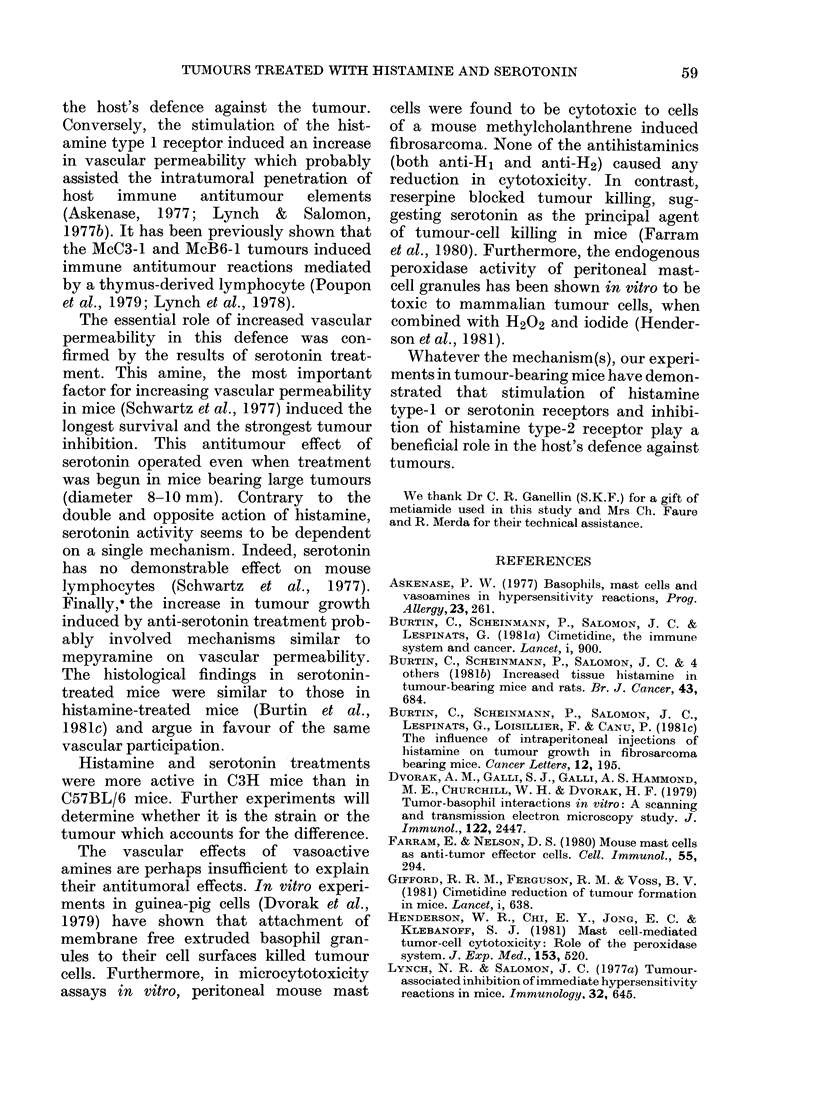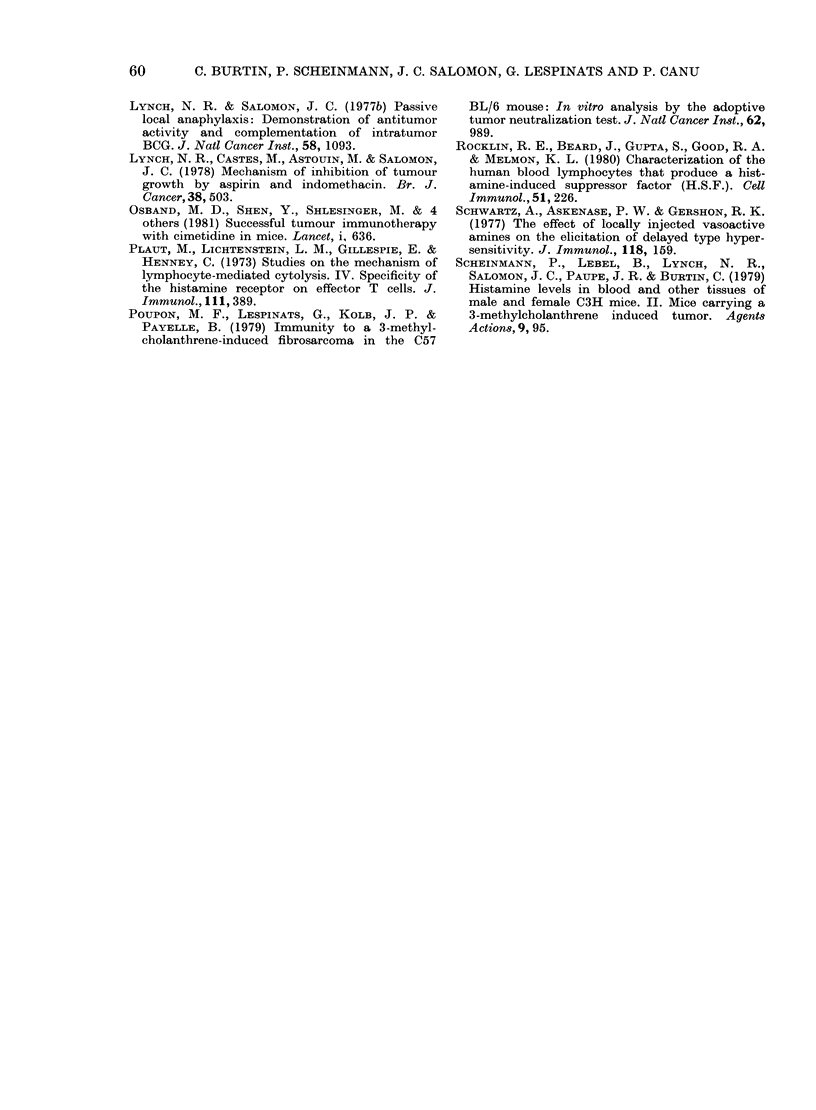# Decrease in tumour growth by injections of histamine or serotonin in fibrosarcoma-bearing mice: influence of H1 and H2 histamine receptors.

**DOI:** 10.1038/bjc.1982.7

**Published:** 1982-01

**Authors:** C. Burtin, P. Scheinmann, J. C. Salomon, G. Lespinats, P. Canu

## Abstract

**Images:**


*Br. J. Cancer* (1982) **45**, 54

### DECREASE IN TUMOUR GROWTH BY INJECTIONS OF HISTAMINE OR SEROTONIN IN FIBROSARCOMA-BEARING MICE: INFLUENCE OF $H_1$ AND $H_2$ HISTAMINE RECEPTORS

C. BURTIN*, P. SCHEINMANN*, J. C. SALOMON†, G. LESPINATS† AND P. CANU*

*From the *U 203 INSERM. Laboratoire de Pathologie Expérimentale, Faculté Necker Enfants Malades, 75730 Paris, Cedex 15 and the †Institut de Recherches Scientifiques sur le Cancer, C.N.R.S. B.P. 8 94802 Villejuif Cedex, France*

Received 27 July 1981 Accepted 8 October 1981

**Summary.—C3H and C57 BL/6 mice carrying methylcholanthrene-induced fibrosarcomas were injected i.p. daily with histamine, metiamide (anti-histamine type-2 receptor), histamine + metiamide, mepyramine (anti-histamine type-1 receptor), serotonin and methysergide (anti-serotonin). Inhibition of tumour growth and lengthened survival were observed with histamine and histamine + metiamide. The best results (both on tumour growth and survival) were obtained with serotonin. Survival was increased by metiamide and decreased by mepyramine and methysergide. In histamine-treated and in serotonin-treated mice, histological studies of the tumours showed large and numerous foci of haemorrhagic necrosis.**

**Stimulation of histamine type-1 or serotonin receptors and inhibition of histamine type-2 receptors play a beneficial role in the host's defence against tumours.**

WE HAVE recently shown that, in mice and rats, the presence of a growing tumour caused increased histamine levels in tissues, even those distant from the tumour. These data were obtained in C3H and C57BL/6 mice bearing a methylcholanthrene-induced fibrosarcoma, in Wag rats bearing an aflatoxin $B_1$-induced hepatoma and in Commentry rats bearing a graft hepatoma (Scheinmann *et al.*, 1979; Burtin *et al.*, 1981*b*).

Despite that increase, the immediate hypersensitivity reactions (passive and active, local and general) were depressed in C3H mice carrying a 3-methylcholanthrene induced fibrosarcoma (McC3-1) (Lynch & Salomon, 1977*a*). These data suggested that the tumour induced a deficit in histamine availability which might favour its growth. Indeed, when this deficit was overcome, the tumour growth was inhibited. This inhibition was obtained by eliciting intratumour passive local anaphylaxis with an extremely high IgE antibody titre, leading to the release of vaso-active amines (Lynch & Salomon, 1977*b*). Furthermore, daily i.p. injections of free histamine significantly inhibited tumour growth in C3H and C57BL/6 mice. Histological studies have shown the presence of numerous large loci of acute haemorrhagic necrosis in tumours of histamine-treated mice (Burtin *et al.*, 1981*c*).

The aim of this work was to study which histamine receptors and which mechanisms were involved in this phenomenon.

### MATERIALS AND METHODS

Histamine dihydrochloride (Prolabo) and serotonin (5-hydroxytryptamine and creatinin sulphate, Prolabo) solutions were neutralized with NaOH and contained 50 mg of histamine 2HCl/ml and 4 mg/ml of serotonin. Metiamide (S.K.F., $H_2$-receptor antagonist), mepyramine (Specia, $H_1$-receptor antagonist) and methysergide (U.M.L. Sandoz, anti-serotonin) were used in solution containing 5 mg/ml, 1 mg/ml and 250 $\mu$g/ml, respectively. Each group of mice received

0·2 ml i.p. of one of these solutions and control groups were injected i.p. with 0·2 ml of saline solution.

Tumour diameters were measured 3× a week. Statistical analysis used the *t* test.

Female C3H and male C57BL/6 (8–16 weeks old) were used throughout the experiments. The colony of mice was initially cesarean-derived and then maintained in a barrier-protected environment.

*C57BL/6 bearing the McB6-1 fibrosarcoma.*—Fibrosarcomas, originally induced by s.c. injection of 2 mg MCA in female C57BL/6 mice and serially transplanted in syngeneic males or females, were used between the 15th and the 25th passages. Tumours were removed aseptically and cut into small pieces which were then stirred in 0·25% trypsin (Difco) in phosphate buffered saline for 90 min at 37°C. The immunogenicity of the McB6-1 tumour was previously demonstrated (Poupon *et al.*, 1979).

Sixty C57BL/6 male mice each received s.c. $10^4$ tumour cells. One day after the cell transfer, mice were randomly divided into 6 equal groups. Each group was injected i.p. 7 days/week with saline solution, histamine base (6 mg), metiamide (1 mg), histamine base (6 mg) + metiamide (1 mg), mepyramine (0·2 mg) and serotonin (0·8 mg).

*C3H mice bearing the McC3-1 fibrosarcoma.*—Fibrosarcomas induced in C3H mice by the i.m. injection of 1 mg 3-methylcholanthrene were maintained by serial transplantation in syngeneic mice and freezing of various passages. The McC3 fibrosarcoma was used between the 15th and 25th passages. Tumour grafts were performed ventrally by the s.c. introduction of small pieces ($\leqslant 1$ mm$^3$) of non-necrotic tumour via a trocar needle. This tumour was immunogenic as described by Lynch *et al.* (1978).

Three different experiments were performed (mice were injected i.p. 5 days/week). Five days after the tumour cell transfer, (1) 10 mice received histamine (6 mg) and 10 mice saline solution and (2) 14 mice received serotonin (0·8 mg) and 10 mice saline solution. In the third experiment, 3 groups of 12 mice received serotonin (0·8), methysergide (50 $\mu$g) or saline solution 20 days after the tumour-cell transfer.

Three serotonin-treated and 3 control C3H mice injected as described in the second experiment, were killed at Day 20 after the tumour-cell transfer. Tumour fragments were fixed in Bouin's fluid, embedded in paraffin, and stained with haematoxylin and eosin.

### RESULTS

### *Histamine treatment in C3H mice*

Histamine significantly inhibited tumour growth for at least 68 days and increased survival (Table I).

### *Histamine and anti-histamines (anti-$H_1$ and anti-$H_2$) treatment in C57BL/6 mice*

Histamine alone significantly inhibited tumour growth for 30 days and increased survival (Table II and Fig. 1). Metiamide alone had no effect on tumour growth, but significantly increased survival (Table II and Fig. 2).

Treatment with histamine and metiamide gave better results than histamine alone for inhibition of tumour growth, but survival was not significantly different (Table II and Fig. 2).

Mepyramine treatment induced a slight but not significant increase in tumour growth and a significant decrease in survival (Table II and Fig. 1).

TABLE I.—*Effect of histamine i.p. on tumour mean diameter (mm ± s.d.) and survival in groups of 10 McC3-1 C3H mice*

| Day | | Control | Histamine |
|---|---|---|---|
| 0 | Injection of tumour cells | + | + |
| 5 | Injection of histamine | − | + |
| 12 | ,, | 4·72 ± 0·26 | 4·13 ± 0·30 |
| 23 | ,, | 8·17 ± 0·68 | 6·00 ± 0·93* |
| 37 | ,, | 16·78 ± 0·51 | 10·44 ± 1·19* |
| 50 | ,, | 26·44 ± 1·60 | 15·14 ± 1·71* |
| 68 | ,, | all dead | 20·30 ± 2·73 |
| 80 | | | 30·67 ± 0·60 (4 survivors) |

* $P < 0{\cdot}01$ by comparison with control mice (*t* test).

TABLE II.—*Evolution of mean tumour diameter* (*mm* ± *s.d.*) *in McB*6-1—*C*57*BL*/6 *mice*

| Day | Control | Histamine | Metiamide | Histamine and Metiamide | Mepyramine |
|---|---|---|---|---|---|
| 15† | + | − | + | − | + |
| 20 | 4·2±0·8 | 2·9±0·4* | 4·7±1·9 | 2·9±1·0* | 4·6±1·7 |
| 25 | 7·9±1·1 | 6·0±0·6* | 8·8±1·3 | 3·0±1·5* | 9·5±1·5 |
| 30 | 12·5±2·7 | 8·6±1·4* | 12·3±2·0 | 8·1±1·8* | 15·0±3·2 |
| 35 | 15·7±2·7 | 12·1±2·1 | 15·1±3·4 | 10·6±2·0* | 18·8±3·1 |
| 40 | 20·3±2·8 | 19·4±4·3 | 22·9±3·8 | 15·7±2·3* | — — |
| 45 | 25·3±2·7 | 23·5±3·3 | 26·8±4·9 | 19·6±2·4* | |
| 50 | 29·9±2·4 | 26·6±3·6 | 30·4±5·3 | 24·3±2·8* | |

Tumour cells were injected at Day 0. At Day 1 each group of 10 mice were injected i.p. daily.

* $P < 0{\cdot}05$ by comparison with control mice ($t$ test).

† Tumour palpable (+) or not (−).

FIG. 1.—Survival curves of McB6-1 C57BL/6 mice. Day 0: injection of tumour cells. Day 1: beginning of i.p. injections of saline solution, ——; histamine, ---; mepyramine, –·–·–.

FIG. 2.—Survival curves of McB6-1 C57BL/6 mice. Day 0: injection of tumour cells. Day 1: beginning of i.p. injections of saline solution, ——; metiamide, –·–·–; histamine + metiamide, ---.

*Serotonin and anti-serotonin treatment of C57BL/6 and C3H mice*

C57BL/6 mice. Serotonin induced a significant decrease in tumour growth for 40 days and an increase in survival. At the end of treatment (Day 50), 4 controls and 9 serotonin treated mice survived. At Day 60, all the controls were dead and 5 treated mice survived (Table III).

C3H mice treated 5 days after tumour-cell transfer.

Tumours grew in all the control mice. The study of serotonin-treated mice revealed 2 distinct populations. In 7 mice, serotonin induced a significant decrease in tumour growth until Day 45 (Table III). In 6 mice, the tumour never developed, and these mice were still alive 4 months after the end of the treatment. In one mouse, the tumour began to develop at Day 16, reached a diameter of 11 mm at Day 25, completely disappeared from Day 44 to Day 70, and grew again in spite of the treatment. Survival was significantly increased (Fig. 3).

C3H mice treated 20 days after tumour-cell transfer.

In serotonin-treated mice, inhibition of tumour growth first appeared 15 days after the beginning of the treatment. This inhibition was still significant ($P < 0{\cdot}05$) at the end of the treatment (Day 70 after

TABLE III.—*Evolution of mean tumour diameter (mm ± s.d.) in McB6-1 C57BL/6 mice (tumour cells injected at day 0 and serotonin begun at Day 1) and in McC3-1 C3H mice (tumour cells injected at day 0 and serotonin begun at Day 5)*

| | C57BL/6 | | C3H | |
|---|---|---|---|---|
| Day | Control (n = 10) | Serotonin (n = 10) | Control (n = 10) | Serotonin (n = 14)† |
| 15 | | | 5·6 ± 1·1 | 4·6 ± 1·3 |
| 20 | 4·2 ± 0·8 | 2·5 ± 0·9** | 8·3 ± 1·1 | 6·4 ± 1·6* |
| 25 | 7·9 ± 1·1 | 3·8 ± 1·2** | 10·1 ± 1·8 | 8·1 ± 1·5* |
| 30 | 12·5 ± 2·7 | 8·1 ± 2·3** | 11·8 ± 2·1 | 9·1 ± 1·5* |
| 35 | 15·7 ± 2·7 | 11·6 ± 3·0** | 14·8 ± 3·0 | 11·4 ± 1·2* |
| 40 | 20·3 ± 2·8 | 15·7 ± 3·4* | 16·5 ± 3·0 | 13·3 ± 2·0* |
| 45 | 25·3 ± 2·7 | 19·8 ± 6·7* | 18·4 ± 2·8 | 15·3 ± 2·1* |
| 50 | 29·9 ± 2·4 | 25·5 ± 7·7 | 19·8 ± 2·7 | 18·0 ± 2·2 |

† Means of 7 tumours, in the other 7 mice, tumour growth was absent or transient (see text).
* $P < 0{\cdot}05$.
** $P < 0{\cdot}01$ by comparison with control mice ($t$ test).

the tumour-cell transfer) and all 12 mice survived.

In methysergide-treated mice, a slight but non-significant increase in tumour growth was observed. However, as in mepyramine-treated mice, the survival decreased: at Day 70, only 25% survived, while in control mice 66% survived.

### *Histology*

Histological examination showed the fibroscarcoma type of the tumour (Fig. 4). C3H mice treated with serotonin had a modified histological structure of the tumours. This consisted mostly of the presence of bands and foci of necrotic and haemorrhagic tissue. These lesions were observed in the central part of tumours (Fig. 5) as well as at the periphery (Fig. 6). This resembled acute necrosis, associated or not with a haemorrhagic phenomenon. No cellular infiltrates surrounded the necrotic areas.

FIG. 3.—Survival curves of McC3-1 C3H mice. Day 0: injection of tumour cells. Day 5: beginning of i.p. injections of saline solution, ——; serotonin, – – – –.

FIG. 4.—Typical histological picture of a McC3-1 fibrosarcoma in a C3H mouse. H. & E. Original magnification. × 150.

FIG. 5.—McC3-1 fibrosarcoma in a serotonin-treated C3H mouse. H. & E. Original magnification × 150. Lesions in the central part of the tumour.

FIG. 6.—McC3-1 fibrosarcoma in a serotonin-treated C3H mouse. H & E. Original magnification × 150. Lesions in the periphery of the tumour.

DISCUSSION

Our results confirm the inhibition by i.p. injections of histamine of tumour growth in C3H and C57BL/6 mice bearing methylcholanthrene-induced fibrosarcomas (Burtin *et al.*, 1981*c*). Histamine also lengthened the survival of tumour-bearing mice.

The analysis of the histamine receptors involved was performed with histamine-receptor antagonists. I.p. injections of metiamide, a histamine type 2-receptor antagonist did not influence tumour growth, but significantly lengthened the survival of tumour-bearing C57BL/6 mice. Longer survival has also been found with oral cimetidine (another histamine type 2-receptor antagonist) in C57BL/6 mice injected with 3 LL tumour cells (Osband *et al.*, 1981) and in the C57BL/6-EL4 and C3H-Mc43 tumour models (Gifford *et al.*, 1981). The inhibiting effect of cimetidine on tumour growth was attributed to the inhibition of suppressor cells. In our experiments, the simultaneous use of histamine and metiamide gave better results than either histamine or metiamide alone on tumour growth (Burtin *et al.*, 1981*a*). Since histamine has been shown to exert an immuno-inhibitory role through the stimulation of the type-2 receptors on T lymphocytes (Plaut *et al.*, 1973; Rocklin *et al.*, 1980) the beneficial effects of histamine alone should be attributed to the stimulation of the histamine type-1 receptors. This fact was confirmed by the increased mortality during treatment with mepyramine (a histamine type 1-receptor antagonist) treatment. Mepyramine probably favoured tumour growth by adding its pharmacological effects (decrease of vascular permeability) to the decrease in histamine availability induced by the tumour itself. Indeed, Lynch & Salomon (1977*b*) have shown that the i.v. injection of a McC3-1 acellular extract in normal mice inhibited the passive cutaneous anaphylaxis reactions. This experiment suggested an "antihistaminic" activity by the tumour. Mepyramine increased this antihistaminic action and thus decreased the host's defence against the tumour. Conversely, the stimulation of the histamine type 1 receptor induced an increase in vascular permeability which probably assisted the intratumoral penetration of host immune antitumour elements (Askenase, 1977; Lynch & Salomon, 1977*b*). It has been previously shown that the McC3-1 and McB6-1 tumours induced immune antitumour reactions mediated by a thymus-derived lymphocyte (Poupon *et al.*, 1979; Lynch *et al.*, 1978).

The essential role of increased vascular permeability in this defence was confirmed by the results of serotonin treatment. This amine, the most important factor for increasing vascular permeability in mice (Schwartz *et al.*, 1977) induced the longest survival and the strongest tumour inhibition. This antitumour effect of serotonin operated even when treatment was begun in mice bearing large tumours (diameter 8–10 mm). Contrary to the double and opposite action of histamine, serotonin activity seems to be dependent on a single mechanism. Indeed, serotonin has no demonstrable effect on mouse lymphocytes (Schwartz *et al.*, 1977). Finally, the increase in tumour growth induced by anti-serotonin treatment probably involved mechanisms similar to mepyramine on vascular permeability. The histological findings in serotonin-treated mice were similar to those in histamine-treated mice (Burtin *et al.*, 1981*c*) and argue in favour of the same vascular participation.

Histamine and serotonin treatments were more active in C3H mice than in C57BL/6 mice. Further experiments will determine whether it is the strain or the tumour which accounts for the difference.

The vascular effects of vasoactive amines are perhaps insufficient to explain their antitumoral effects. *In vitro* experiments in guinea-pig cells (Dvorak *et al.*, 1979) have shown that attachment of membrane free extruded basophil granules to their cell surfaces killed tumour cells. Furthermore, in microcytotoxicity assays *in vitro*, peritoneal mouse mast cells were found to be cytotoxic to cells of a mouse methylcholanthrene induced fibrosarcoma. None of the antihistaminics (both anti-$H_1$ and anti-$H_2$) caused any reduction in cytotoxicity. In contrast, reserpine blocked tumour killing, suggesting serotonin as the principal agent of tumour-cell killing in mice (Farram *et al.*, 1980). Furthermore, the endogenous peroxidase activity of peritoneal mast-cell granules has been shown *in vitro* to be toxic to mammalian tumour cells, when combined with $H_2O_2$ and iodide (Henderson *et al.*, 1981).

Whatever the mechanism(s), our experiments in tumour-bearing mice have demonstrated that stimulation of histamine type-1 or serotonin receptors and inhibition of histamine type-2 receptor play a beneficial role in the host's defence against tumours.

We thank Dr C. R. Ganellin (S.K.F.) for a gift of metiamide used in this study and Mrs Ch. Faure and R. Merda for their technical assistance.

### REFERENCES

Askenase, P. W. (1977) Basophils, mast cells and vasoamines in hypersensitivity reactions, *Prog. Allergy*, **23**, 261.

Burtin, C., Scheinmann, P., Salomon, J. C. & Lespinats, G. (1981*a*) Cimetidine, the immune system and cancer. *Lancet*, i, 900.

Burtin, C., Scheinmann, P., Salomon, J. C. & 4 others (1981*b*) Increased tissue histamine in tumour-bearing mice and rats. *Br. J. Cancer*, **43**, 684.

Burtin, C., Scheinmann, P., Salomon, J. C., Lespinats, G., Loisillier, F. & Canu, P. (1981*c*) The influence of intraperitoneal injections of histamine on tumour growth in fibrosarcoma bearing mice. *Cancer Letters*, **12**, 195.

Dvorak, A. M., Galli, S. J., Galli, A. S. Hammond, M. E., Churchill, W. H. & Dvorak, H. F. (1979) Tumor-basophil interactions *in vitro*: A scanning and transmission electron microscopy study. *J. Immunol.*, **122**, 2447.

Farram, E. & Nelson, D. S. (1980) Mouse mast cells as anti-tumor effector cells. *Cell. Immunol.*, **55**, 294.

Gifford, R. R. M., Ferguson, R. M. & Voss, B. V. (1981) Cimetidine reduction of tumour formation in mice. *Lancet*, i, 638.

Henderson, W. R., Chi, E. Y., Jong, E. C. & Klebanoff, S. J. (1981) Mast cell-mediated tumor-cell cytotoxicity: Role of the peroxidase system. *J. Exp. Med.*, **153**, 520.

Lynch, N. R. & Salomon, J. C. (1977*a*) Tumour-associated inhibition of immediate hypersensitivity reactions in mice. *Immunology*, **32**, 645.

LYNCH, N. R. & SALOMON, J. C. (1977*b*) Passive local anaphylaxis: Demonstration of antitumor activity and complementation of intratumor BCG. *J. Natl Cancer Inst.*, **58**, 1093.

LYNCH, N. R., CASTES, M., ASTOUIN, M. & SALOMON, J. C. (1978) Mechanism of inhibition of tumour growth by aspirin and indomethacin. *Br. J. Cancer*, **38**, 503.

OSBAND, M. D., SHEN, Y., SHLESINGER, M. & 4 others (1981) Successful tumour immunotherapy with cimetidine in mice. *Lancet*, i, 636.

PLAUT, M., LICHTENSTEIN, L. M., GILLESPIE, E. & HENNEY, C. (1973) Studies on the mechanism of lymphocyte-mediated cytolysis. IV. Specificity of the histamine receptor on effector T cells. *J. Immunol.*, **111**, 389.

POUPON, M. F., LESPINATS, G., KOLB, J. P. & PAYELLE, B. (1979) Immunity to a 3-methylcholanthrene-induced fibrosarcoma in the C57 BL/6 mouse: *In vitro* analysis by the adoptive tumor neutralization test. *J. Natl Cancer Inst.*, **62**, 989.

ROCKLIN, R. E., BEARD, J., GUPTA, S., GOOD, R. A. & MELMON, K. L. (1980) Characterization of the human blood lymphocytes that produce a histamine-induced suppressor factor (H.S.F.). *Cell Immunol.*, **51**, 226.

SCHWARTZ, A., ASKENASE, P. W. & GERSHON, R. K. (1977) The effect of locally injected vasoactive amines on the elicitation of delayed type hypersensitivity. *J. Immunol.*, **118**, 159.

SCHEINMANN, P., LEBEL, B., LYNCH, N. R., SALOMON, J. C., PAUPE, J. R. & BURTIN, C. (1979) Histamine levels in blood and other tissues of male and female C3H mice. II. Mice carrying a 3-methylcholanthrene induced tumor. *Agents Actions*, **9**, 95.